# High prevalence of Chlamydia trachomatis infection among women attending STD and gynecology clinics in Jiangsu province, China: A cross-sectional survey

**DOI:** 10.1097/MD.0000000000027599

**Published:** 2021-11-19

**Authors:** Haiyang Hu, Ying Zhou, Lingen Shi, Jing Lu, Zhi Zhang, Xiaoqin Xu, Xiping Huan, Gengfeng Fu

**Affiliations:** Section of STD/AIDS Control and Prevention, Jiangsu Provincial Center for Disease Control and Prevention, Nanjing, China.

**Keywords:** China, Chlamydia trachomatis, gynecology clinic, prevalence, STD clinic, women

## Abstract

Epidemics of Chlamydia trachomatis (CT) infection among women are major global public health concerns. This study examined the prevalence of CT infection and associated factors among women attending sexually transmitted disease (STD) and gynecology clinics in Jiangsu province, China.

A cross-sectional survey was conducted among women attending STD and gynecology clinics in the province during 2018 to 2019. Socio-demographic and behavioral information were collected through face-to-face questionnaire survey. Cervical swab specimens were collected to test for CT. Chi square tests were used to compare differences in CT prevalence between subgroups of characteristics. Multivariate logistic regression analysis was used to identify factors associated with CT infection.

A total of 2664 participants were enrolled. The prevalence of CT infection was 16.6% (95% confidence interval: 15.0%–18.1%). Of those, CT prevalence among participants from STD clinics (19.4%) and South Jiangsu (18.5%) were higher. Female outpatients who were service personnel (adjusted odds ratio [aOR] = 1.667, *P* = .004) or farmers (aOR = 1.593, *P* = .039), lived in South Jiangsu (aOR = 1.796, *P* = .004), and were from STD clinics (aOR = 1.608, *P* = .022) were more likely to infect CT.

Our study showed a high prevalence of CT infection among women attending STD and gynecology clinics in Jiangsu province, China. CT screening, surveillance and treatment promotion should therefore be of top priority on the CT prevention agenda.

## 1 Introduction

Sexually transmitted diseases (STDs) are among the most common infectious diseases globally and significantly associated with morbidity and mortality worldwide. According the World Health Organization, there were an estimated 376.4 million infections of the 4 primary STDs (syphilis, chlamydia, gonorrhea, and trichomoniasis) in 2016, of which 127.2 million chlamydia infections.^[1]^ Genital chlamydia infection (caused by *Chlamydia trachomatis* [CT]) is known to cause pelvic inflammatory disease in women, increase the risk for tubal factor infertility, ectopic pregnancy, miscarriage, and preterm birth.^[2–4]^ In addition, CT infections can increase the risk of human immunodeficiency virus (HIV) infection, and among people living with HIV, can increase the risk of transmitting HIV to sexual partners.^[5]^ Among pregnant women living with HIV, it also can increase the risk of HIV transmission to infants.^[6]^

According to a systematic review from 100 studies,^[1]^ the global estimated prevalence rate of CT in 2016 was approximately 3.8% among women aged 15 to 49 years. The World Health Organization American region had the highest prevalence (7.0%), while the South-East Asia region had the lowest (1.5%). Of these 100 studies, there were only 4 Chinese studies,^[7–10]^ including attendees at an obstetrics and gynecology clinic from Wuhan with a prevalence of 5.4%,^[7]^ and attendees at maternity clinics from 16 eastern cities with a 1.5% prevalence.^[8]^

As early as in 2001, U.S. Preventive Services Task Force recommended screening sexually active adolescents and adults for chlamydia infection.^[11]^ In 2007, the U.S. Preventive Services Task Force updated this recommendation. Several category suggestions were made according to different characteristics (e.g., gender, age, pregnancy or not, sexual activity or not).^[12]^ In contrast, there was no systematic advices in China. Jiangsu province, is an economically developed region with more than 80 million people, located in the southeastern part of China. Towards the end of 2020, few findings about CT prevalence had been reported. Herein, we conducted this cross-sectional survey to examine the prevalence of CT, identify factors associated with CT infection among female patients attending STD and gynecology clinics in Jiangsu province, China, and provide data for policy making.

## 2 Materials and methods

### 2.1 Study design and participants

A cross-sectional survey was conducted at the STD surveillance sites throughout Jiangsu province during 2018 to 2019. The sample size calculated was approximately 2200 with PASS software (version 15, NCSS, LLC). Participating patients were from STD and gynecology clinics (see the Acknowledgements section for detailed sources). Only females aged 18 to 60 years old, and had urinary or genital tract symptoms were eligible for inclusion. Each recruited participant had a questionnaire interview and cervical swab collection. All participants provided written informed consent before enrolment. Implementation of this survey and data analysis were approved by Institutional Review Board of National Center for AIDS/STD Control and Prevention, Chinese Center for Disease Control and Prevention (#X170113431).

### 2.2 Measures

Simple random sampling method was used to recruit participants. After the regular visits by doctors, participants were interviewed face-to-face by trained interviewers. Throughout the study, the interviews were anonymous and we were not able to obtain information that could identify individual participants. The information collected using the questionnaire included socio-demographic information (age, marital status, education level, registered residence, and occupation), and CT related behavioral data (number of pregnancies, contraception method, age of first sexual behavior, frequency of sexual behavior, and number of sexual partners in the past 6 months).

Cervical swabs collected from each participant were tested for CT. All specimens were temporarily stored at –20°C in the local laboratory for approximately 1 month, and later transported to the Jiangsu Provincial Center for Disease Control and Prevention (Jiangsu Center for Disease Control and Prevention) for testing. Nucleic acid amplification testing method was used to detect CT DNA with a commercialized reagent (Jiangsu Bioperfectus Technologies Co., Ltd., Taizhou, China; Catalog Number: JB60102N). First, the swabs were soaked in the saline in order to get the cells. Second, DNA was extracted from the saline including cells with the magnetic bead method. Finally, real-time polymerase chain reaction tests were conducted to detect CT DNA with extracted DNA as template. If tests were positive, participants were considered as CT infections.

### 2.3 Statistical analysis

Questionnaire data were double-entered and checked for accuracy using Epi data software (version 3.1, Epi Data Association, Odense, Denmark). Participants missing important variables were disqualified and not included in statistical analyses. According to the distributions of data, the quantitative variables were grouped and part of the qualitative variables were merged. Frequency analyses were used to show socio-demographic and behavioral characteristics of participants. Chi square tests were used to compare differences of CT prevalence between subgroups of characteristics. Factors associated with CT infection were first assessed using univariate logistic regression analysis. Variables with *P* values less than .20 were entered into multivariable logistic regression models. Multivariable logistic regression analyses were conducted using forward method in order to determine the adjusted odds ratios (aORs). *P* values less than .05 were considered statistically significant. All analyses were conducted using SPSS software (version 19.0, SPSS Inc., Chicago).

## 3 Results

### 3.1 Socio-demographic characteristics of participants

Overall, 2664 eligible patients were enrolled into the study according to the questionnaire and the test. Of those, 29.5% (785) were from STD clinics and 70.5% (1879) from gynecology clinics. The average age of participants was 35.4 with a standard deviation of 9.3 (range: 18–60). Participants aged 18–29 years constituted 32.6% (868), and those aged 30–39 years constituted 35.6% (949). Majority of participants (90.1%, 2308) were married or cohabiting and approximately half (50.2%, 1270) had an education level of junior high school or lower. 35.5% (857) of participants were factory workers, 22.4% (541) were service personnel, and 14.4% (347) were farmers. 47.2% (1257) lived in South Jiangsu, 34.9% (930) in North Jiangsu and 17.9% (477) in Central Jiangsu (Table 1).

**Table 1:** Socio-demographic and behavioral characteristics of participants and the prevalence of Chlamydia trachomatis infection.

Characteristics	N (%)	CT cases	Prevalence (%) (95% CI)	*P* value
Age (yrs)				.464
18–29	868 (32.6)	145	16.7 (14.0–19.4)	
30–39	949 (35.6)	160	16.9 (14.2–19.5)	
40–49	601 (22.6)	104	17.3 (14.0–20.6)	
50–60	246 (9.2)	32	13.0 (8.5–17.5)	
Marital status^∗^				.006
Single, divorced or widowed	255 (9.9)	57	22.4 (16.5–28.2)	
Married or cohabiting	2308 (90.1)	362	15.7 (14.1–17.3)	
Education level^∗^				.605
Junior high school or lower	1270 (50.2)	197	15.5 (13.3–17.7)	
Senior high school	715 (28.2)	123	17.2 (14.2–20.2)	
College or higher	546 (21.6)	90	16.5 (13.1–19.9)	
Registered residence^∗^				.017
Jiangsu province	2369 (90.0)	377	15.9 (14.3–17.5)	
Other provinces	263 (10.0)	57	21.7 (16.0–27.3)	
Occupation^∗^				.005
Factory worker	857 (35.5)	117	13.7 (11.2–16.1)	
Farmer	347 (14.4)	61	17.6 (13.2–22.0)	
Service personnel	541 (22.4)	115	21.3 (17.4–25.1)	
Office clerk	169 (7.0)	21	12.4 (7.1–17.7)	
Individual operator	286 (11.9)	51	17.8 (12.9–22.7)	
Others or unemployment	213 (8.8)	38	17.8 (12.2–23.5)	
Sub-district				.004
South Jiangsu	1257 (47.2)	233	18.5 (16.2–20.9)	
North Jiangsu	930 (34.9)	151	16.2 (13.6–18.8)	
Central Jiangsu	477 (17.9)	57	11.9 (8.8–15.1)	
Clinics				.012
STD	785 (29.5)	152	19.4 (16.3–22.4)	
Gynecology	1879 (70.5)	289	15.4 (13.6–17.2)	
Number of pregnancies^∗^				.049
None	373 (14.4)	69	18.5 (14.1–22.9)	
One time	1063 (41.1)	182	17.1 (14.6–19.6)	
Two times	785 (30.4)	104	13.2 (10.7–15.8)	
Three or more times	364 (14.4)	65	17.9 (13.5–22.2)	
Contraception method^∗^				.892
Condom	666 (28.3)	113	17.0 (13.8–20.1)	
Others^†^	1691 (71.7)	283	16.7 (14.8–18.7)	
Age of first sexual behavior (years)^∗^				.003
15–19	262 (10.4)	61	23.3 (17.4–29.1)	
20–24	1999 (79.5)	309	15.5 (13.7–17.2)	
≥25	255 (10.1)	50	19.6 (14.2–25.0)	
Frequency of sexual behavior^∗^				.599
Once 2 wks or less or none	948 (39.0)	156	16.5 (13.9–19.0)	
Approximately once a week	907 (37.3)	137	15.1 (12.6–17.6)	
Twice a week or more	574 (23.6)	97	16.9 (13.5–20.3)	
Number of sexual partners, past 6 months^∗^				.106
One	1878 (91.6)	311	16.6 (14.7–18.4)	
Two or more	173 (8.4)	37	21.4 (14.5–28.3)	

CI = confidence interval, CT = Chlamydia trachomatis, STD = sexually transmitted disease.

∗ Data missing or refusal.

† Other contraception methods refer to those other than condom, including oral pill, intrauterine devices (IUD), injectable, and vaginal ring.

### 3.2 Prevalence of CT

**Figure 1:**
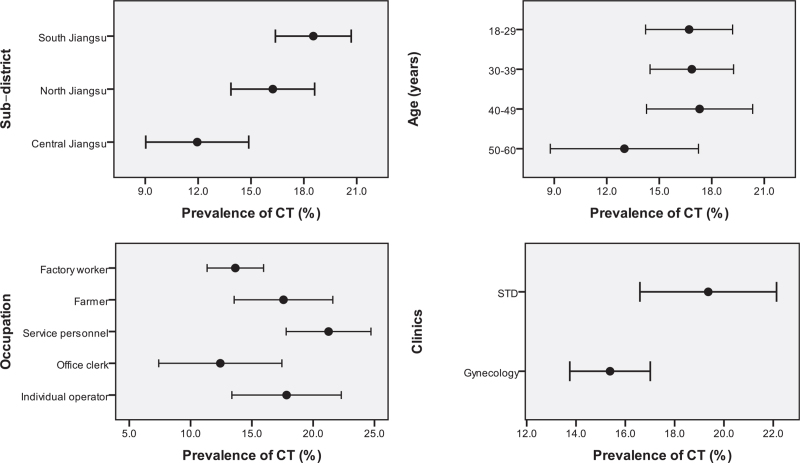
Differences in the prevalence of Chlamydia trachomatis (CT) infection between subgroups of 4 main variables. The 4 variables were sub-district, age, occupation and clinics, respectively. Of those, there were significant differences in CT prevalence between sub-district, occupations, and clinics, and there was no significant difference between age groups.

Among 2664 participants, 441 tested positive for CT infection, showing a high prevalence of 16.6% (95% confidence interval [CI]: 15.0%–18.1%). Of those, the prevalence of CT infection of patients from STD clinics was 19.4% (95% CI: 16.3%–22.4%), higher than that from gynecology clinics (15.4%, 95% CI: 13.6%–17.2%, *P* = .012). We observed a significant difference in CT prevalence by the different regions of the province (*P* = .004), with 18.5% (95% CI: 16.2%–20.9%) in South Jiangsu, 16.2% (95% CI: 13.6%–18.8%) in North Jiangsu, and 11.9% (95% CI: 8.8%–15.1%) in Central Jiangsu, respectively. Besides, CT prevalence among participants being single, divorced, or widowed (22.4%, 95% CI: 16.5%–28.2%, *P* = .006), and being registered residence of other provinces (21.7%, 95% CI: 16.0%–27.3%, *P* = .017), and with occupation of service personnel (21.3%, 95% CI: 17.4%–25.1%, *P* = .005) were higher. There was no significant difference in CT prevalence between age groups (Table 1 and Fig. 1).

### 3.3 Factors associated with CT infection

The potential factors associated with CT infection were analyzed with CT negative patients as control. In the univariate analysis, CT infection was significantly associated with marital status, registered residence, occupation, sub-district, clinic category, and number of pregnancies (Table 2). In the multivariate analysis, female outpatients who were service personnel (aOR = 1.667, 95% CI: 1.178–2.359, *P* = .004) or farmers (aOR = 1.593, 95% CI: 1.023–2.480, *P* = .039), lived in South Jiangsu (aOR = 1.796, 95% CI: 1.204–2.680, *P* = .004), and were from STD clinics (aOR = 1.608, 95% CI: 1.071–2.415, *P* = .022) were more likely to infect CT (Table 2).

**Table 2:** Univariate and multivariate analysis of factors associated with CT infection among women attending STD and gynecology clinics.

Variable	Negative N (%)	Positive N (%)	OR (95% CI)	*P* value	aOR (95% CI)	*P* value
Age (yrs)						
18–29	723 (32.5)	145 (32.9)	1.341 (0.888–2.025)	0.163	1.095 (0.621–1.930)	.755
30–39	789 (35.5)	160 (36.3)	1.356 (0.901–2.040)	0.144	1.092 (0.637–1.874)	.749
40–49	497 (22.4)	104 (23.6)	1.399 (0.913–2.146)	0.123	1.109 (0.640–1.923)	.712
50–60	214 (9.6)	32 (7.3)	1		1	
Marital status						
Single, divorced or widowed	198 (9.2)	57 (13.6)	1.548 (1.129–2.121)	0.007	1.187 (0.753–1.873)	.461
Married or cohabiting	1946 (90.8)	362 (86.4)	1		1	
Education level						
Junior high school or lower	1073 (50.6)	197 (48.0)	1			
Senior high school	592 (27.9)	123 (30.0)	1.132 (0.884–1.448)	0.326		
College or higher	456 (21.5)	90 (22.0)	1.075 (0.819–1.412)	0.603		
Registered residence						
Jiangsu province	1992 (90.6)	377 (86.9)	1		1	
Other provinces	206 (9.4)	57 (13.1)	1.462 (1.069–2.000)	0.017	1.472 (0.982–2.207)	.061
Occupation						
Factory worker	740 (36.8)	117 (29.0)	1		1	
Farmer	286 (14.2)	61 (15.1)	1.349 (0.962–1.892)	0.083	1.593 (1.023–2.480)	.039
Service personnel	426 (21.2)	115 (28.5)	1.707 (1.286–2.267)	0.000	1.667 (1.178–2.359)	.004
Office clerk	148 (7.4)	21 (5.2)	0.897 (0.546–1.475)	0.669	0.979 (0.544–1.764)	.945
Individual operator	235 (11.7)	51 (12.7)	1.373 (0.958–1.968)	0.085	1.184 (0.753–1.862)	.465
Others or unemployment	175 (8.7)	38 (9.4)	1.373 (0.919–2.052)	0.121	1.242 (0.736–2.095)	.417
Sub-district						
South Jiangsu	1024 (46.1)	233 (52.8)	1.677 (1.228–2.288)	0.001	1.796 (1.204–2.680)	.004
North Jiangsu	779 (35.0)	151 (34.2)	1.428 (1.030–1.981)	0.033	0.976 (0.609–1.567)	.921
Central Jiangsu	420 (18.9)	57 (12.9)	1		1	
Clinics						
STD	633 (28.5)	152 (34.5)	1.321 (1.063–1.641)	0.012	1.608 (1.071–2.415)	.022
Gynecology	1590 (71.5)	289 (65.5)	1		1	
Number of pregnancies						
None	304 (14.0)	69 (16.4)	1.044 (0.718–1.518)	0.821	1.254 (0.724–2.171)	.418
One time	881 (40.7)	182 (43.3)	0.950 (0.696–1.298)	0.749	1.041 (0.697–1.554)	.844
Two times	681 (31.5)	104 (24.8)	0.702 (0.501–0.985)	0.041	0.809 (0.534–1.227)	.320
Three or more times	299 (13.8)	65 (15.5)	1		1	
Contraception method						
Condom	553 (28.2)	113 (28.5)	1			
Others	1408 (71.8)	283 (71.5)	0.984 (0.774–1.250)	0.892		
Age of first sexual behavior (yrs)						
15–19	201 (9.6)	61 (14.5)	1.244 (0.816–1.897)	0.309	1.221 (0.687–2.170)	.496
20–24	1690 (80.6)	309 (73.6)	0.750 (0.538–1.045)	0.089	0.917 (0.591–1.423)	.699
≥25	205 (9.8)	50 (11.9)	1		1	
Frequency of sexual behavior						
Once 2 wks or less or none	792 (38.8)	156 (40.0)	1			
Approximately once a week	770 (37.8)	137 (35.1)	0.903 (0.703–1.160)	0.425		
Twice a week or more	477 (23.4)	97 (24.9)	1.032 (0.782–1.363)	0.822		
Number of sexual partners, past 6 months						
One	1567 (92.0)	311 (89.4)	1		1	
Two or more	136 (8.0)	37 (10.6)	1.371 (0.934–2.011)	0.107	0.668 (0.411–1.086)	.104

aOR = adjusted odds ratio, CI = confidence interval, CT = Chlamydia trachomatis, OR = odds ratio, STD = sexually transmitted disease.

## 4 Discussion

Though CT infection has become a major public health and social problems, study data sets on the subject are still limited in China. Our study is among the first to examine CT prevalence among female outpatients in Jiangsu province of China with a large sample size. We observed a CT prevalence of more than 16%, with higher prevalence among STD clinic patients and lower prevalence among gynecology clinic patients. Our observed prevalence was significantly higher than that reported in Shenzhen (5.4% in 2012),^[7]^ Shaanxi (3.4%),^[9]^ and Beijing (2.2%),^[10]^ and higher than that in Shenzhen (10.3% in 2018).^[13]^ This difference might be associated with the admittance criteria of our study participants as we required that eligible participants had urinary or genital tract symptoms. Nonetheless, our observed prevalence was similar to results reported abroad, slightly higher than that reported in Spain (12.3%),^[14]^ Iran (12.6%),^[15]^ and Mexico (14.2%),^[16]^ and lower than that reported in Ethiopia (18.9%)^[17]^ and Palestine (20.2%).^[18]^ Besides, the fact that many CT infections do not have any related symptoms cannot be ignored.^[1]^

Consistent with most studies,^[16–18]^ education level was not a significant risk factor of CT infection in our study. This could be because the rapid development of information technology and the popularization of the Internet have made comprehensive knowledge of STD prevention easily attainable through a variety of new media, including the most used electronic images and videos. In the past, comprehensive knowledge of STD prevention was popularized using traditional media such as books and folding posters. These were more accessible to people with higher education. As a result, those with low education level might be lower in understanding the knowledge of CT prevention, and thus had higher risk of CT infection. Therefore, the influence of education on CT infection had gradually subsided.

According to the multivariate analysis, marital status and registered residence were not significantly associated with CT infection, but being single, divorced, or widowed and registered residence of other provinces were potential risk factors of CT infection in the univariate analysis. Consistent with our findings, a study in Shenzhen reported that single, divorced, or widowed and migrant women were more likely to have unprotected sexual behavior, and thus more likely to infect CT.^[13]^

In line with a previous literature,^[13]^ our study found CT prevalence to be higher in STD clinic patients than in gynecology clinic patients. This could be due to the reasonable assumption that outpatients of STD clinics had riskier sexual behaviors. Similarly, outpatients with occupations of service personnel and farmer were more likely to infect CT. In addition to unsafe sex, unhealthy hygiene practices might also be to blame. There might be a small number of participants may be involved in sex worker in addition to their occupation as service personnel.

Findings from many studies have shown that there were regional differences in CT infection prevalence.^[1,19–23]^ This is also a basic feature of infectious diseases. Globally, the prevalence of CT infection was highest in the upper middle-income countries and regions.^[1,19]^ In our study, the CT prevalence was significantly higher in South Jiangsu than in the other 2 regions. As South Jiangsu is the most economically developed region in the province, there were 2 possible reasons for this observation. First, extramarital sexual behaviors and multiple sexual partners were more likely to occur. Second, because of better medical resources and higher incomes, women with symptoms or risks of CT infection in this region were likely to be well educated and mobilized enough to get CT tests.^[24,25]^

Through this study, we obtained the prevalence of CT infection among women attending STD and gynecology clinics in Jiangsu province and possible risk factors, which is very important for future chlamydia control. However, several limitations of this study should be noted. First, participants might provide incorrect, fragmentary, or socially desirable answers due to memory bias and face-to-face interview survey mode. Second, other factors that may affect CT infection, such as partners’ infection status, extramarital sex, and other STDs, were not recorded in the current study. Future studies may add these factors into their designs. Third, our study was conducted among women attending STD and gynecology clinics in Jiangsu province, and our results may not be generalizable to all women in the province and other regions.

## 5 Conclusions

In conclusion, this study demonstrates a high prevalence of CT infection among women attending STD and gynecology clinics in Jiangsu province, China. CT screening, surveillance and treatment promotion should therefore be of top priority on the CT prevention agenda.

## Acknowledgments

We thank all staff members of the STD surveillance hospitals for their dedicated work. They are from Nanjing Luhe Center for Maternal and Child Health, Yixing Center for Maternal and Child Health, Yixing Dermatology Disease Prevention and Treatment Institute, Changzhou Jintan People's Hospital, Qidong People's Hospital, Qidong Hospital of Traditional Chinese Medicine, Lianyungang Dongfang Hospital, The 149th Hospital of the Chinese People's Liberation Army, Huai’an District Dermatology Prevention and Treatment Hospital, Sheyang Center for Disease Control and Prevention, Sheyang Dermatology Disease Prevention and Treatment Institute, Yizheng Center for Disease Control and Prevention, Yizheng Center for Maternal and Child Health, Yangzhong People's Hospital, Yangzhong Hospital of Traditional Chinese Medicine, and Yangzhong Center for Disease Control and Prevention.

## Author contributions

All authors have read and agreed to the published version of the manuscript.

**Conceptualization:** Haiyang Hu, Gengfeng Fu.

**Data curation:** Haiyang Hu, Lingen Shi.

**Formal analysis:** Haiyang Hu, Lingen Shi.

**Funding acquisition:** Gengfeng Fu.

**Investigation:** Ying Zhou, Jing Lu, Zhi Zhang.

**Methodology:** Haiyang Hu, Gengfeng Fu.

**Project administration:** Xiaoqin Xu, Xiping Huan.

**Resources:** Ying Zhou, Jing Lu, Zhi Zhang.

**Supervision:** Xiaoqin Xu, Xiping Huan.

**Writing – original draft:** Haiyang Hu.

**Writing – review & editing:** Gengfeng Fu.
